# Female splenogonadal fusion: A PLEA for conservative management

**DOI:** 10.1016/j.eucr.2023.102620

**Published:** 2023-11-16

**Authors:** Silvia Ceccanti, Giulia Varrasso, Miriam D'Avanzo, Gianmarco Andreoli, Gabriele Masselli, Francesca Tarani, Denis A. Cozzi

**Affiliations:** a Pediatric Surgery Unit, Azienda Ospedaliero Universitaria Policlinico Umberto I, Department of Maternal Infantile and Urological Sciences, Sapienza University of Rome, Viale Regina Elena 324, Rome, RM 00161, Italy; b Pediatric Unit, Azienda Ospedaliero Universitaria Policlinico Umberto I, Department of Maternal Infantile and Urological Sciences, Sapienza University of Rome, Viale Regina Elena 324, Rome, RM 00161, Italy; c Department of Radiological, Oncological and Pathological Sciences, Azienda Ospedaliero Universitaria Policlinico Umberto I, Sapienza University of Rome, Viale Regina Elena 324, Rome, RM 00161, Italy

**Keywords:** Splenic-gonadal fusion, Children, Cryptorchidism, Spleno-ovarian fusion, Infantile hemangioma

## Abstract

Splenogonadal fusion in female patients is seldom reported. We describe a 6-month-old girl who represents the youngest living female with splenogonadal fusion reported to date. The lesion was diagnosed as an incidental finding during screening abdominal ultrasonography performed for a vulvar infantile hemangioma. A tail-like structure with splenic echotexture connecting a normally located spleen and the left ovary was detected and better characterized by MRI. We also reviewed the pertinent literature on managing this usually asymptomatic condition, especially in female patients. Greater professionals’ awareness of this benign anomaly is paramount to avoid the unnecessary removal of an otherwise normal gonad.

## Patient consent statement

Written informed consent was obtained from parents for publication of this manuscript and any accompanying images.

## 1 Introduction

The fusion between splenic and gonadal tissue is a rare congenital anomaly. Medical literature consists primarily of case reports that have been first extensively reviewed in 1956 by Putschar and Manion, who detailed 30 clinical cases and coined the current classification system.^1^ A recent update on the subject counted approximately 200 additional cases.^2^ Notably, females are significantly underrepresented, accounting only for less than one-tenth of all cases reported until now. Herein, we report the youngest living female ever to be diagnosed with splenogonadal fusion, and provide an overview of current literature on the management of this usually asymptomatic condition, which has essentially a benign course, especially in female patients.

## 2 Case report

**Fig. 1:**
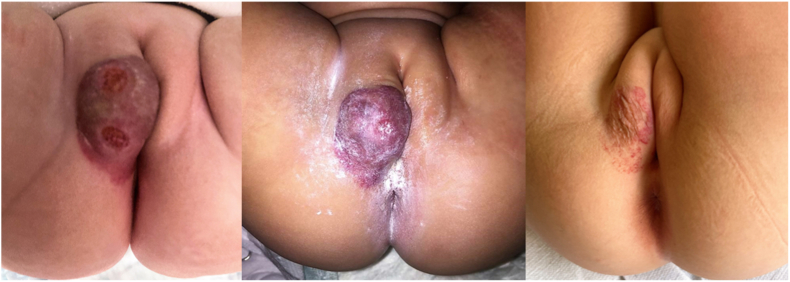
Sequential close-up views of a large vulvar hemangioma involving the right labium majus. Note the ulceration at presentation, with propranolol-induced healing and progressive regression at latest 20 months follow-up.

**Fig. 2:**
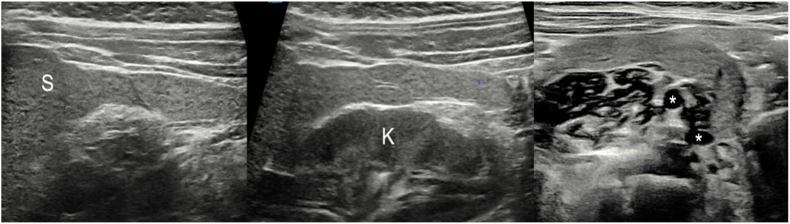
Sequential longitudinal ultrasound images of continuous splenogonadal fusion. Note the tail-like structure with splenic echotexture originating from a normally located spleen (S), traversing the left paracolic gutter laterally to the left kidney (K), and finally embracing the left ovary, which contains small follicles or cysts (asterisks).

A 6-month-old female infant was referred to our clinic for evaluation of an ulcerated infantile vulvar hemangioma (Fig. 1). Requested abdominal ultrasonography did not reveal any particular abnormality of explored intrabdominal organs, apart from a bizarre tail-like structure with splenic echotexture connecting a normally located spleen and the left ovary (Fig. 2). This finding was highly suggestive of splenogonadal fusion of the continuous type.

**Fig. 3:**
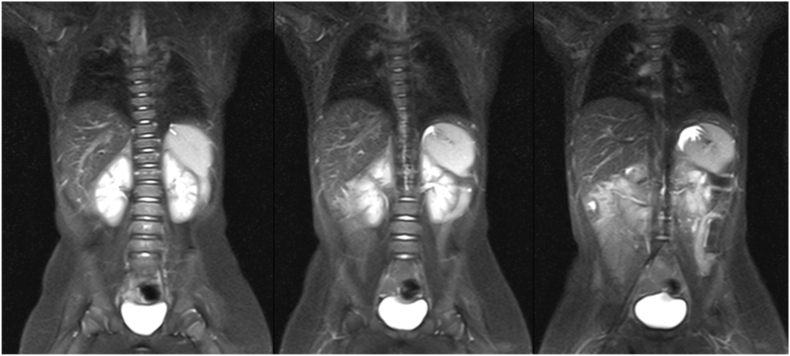
Sequential coronal MRI images of continuous splenogonadal fusion, demonstrating the tail-like structure connecting the normally located spleen and left ovary.

On physical examination, she was otherwise healthy, except for a large hemangioma involving the right labium majus of the vulva, with initial signs of ulceration. Following also a negative cardiac workup, treatment was started with oral propranolol at a dosage of 0.5 mg/kg twice daily, increased to 1 mg/kg twice daily after one week. Propranolol administration induced an effective slowdown of the hemangioma growth and progressive regression of the vascular lesion. At 10 months of age, an abdomen MRI was requested to better characterize the abnormality ultrasonographically detected (Fig. 3). The multidisciplinary team decided to opt for conservative management. Propranolol treatment was withdrawn at 1 year of age because the hemangioma had almost completely regressed. She then regularly attended outpatient appointments including repeat abdominal ultrasonographic examinations, the latest performed when she was 20 months old. Subsequently, the family decided to move abroad permanently, and therefore arrangements were made for a safe and effective patient handover.

## 3 Discussion

Splenogonadal fusion is a rare malformation in which the spleen is abnormally connected to the gonad, or, more rarely, to a derivative of the mesonephros.^1^ The condition has been described only on the left side, reflecting the anatomical proximity of the left gonad and the spleen during embryogenesis. Two varieties of the anomaly are recognized.^1^ In the continuous type, a band of tissue connects the normally located spleen to the gonadal-mesonephric structures. The band may be splenic tissue or fibrous, with splenic nodules at more or less frequent intervals along it. The discontinuous type represents the fusion of accessory splenic tissue and the gonad without a distinct structural connection with the spleen.

Splenogonadal fusion may occur as an isolated condition or with other malformations, which are usually more frequently encountered in the continuous form.^3^ Amongst these, micrognathia and transverse limb reduction defects are rare anomalies that, when present, reflect a disturbance of embryogenesis likely secondary to the intimate topographic adherence of the splenic primordium, the gonadal anlage and other structures derived from the mesonephric ridge, occurring before gonadal descent and mesonephric involution begin. When the gonad descends, it seems to draw out the splenic tissue and produce the splenic cord. In other instances, the descending gonad detaches a portion of the splenic primordium and carries it down.

Sex distribution strongly favors males, with a reported sex ratio ranging from 16.6:1^4^ to 9:1.^1^ Notably, Watson^5^ suggested that such a male excess is biased by the natural sex-based difference in ready accessibility of the gonads during physical examination. Therefore, Gouw et al.^3^ calculated that, after excluding all males presenting with cryptorchidism or with another apparent inguinal or scrotal swelling, the sex ratio decreased to 4:1.

Nevertheless, splenogonadal fusion female cases are undoubtedly underrepresented or underreported. We counted a total of 14 cases previously reported. Of these, nine had severe associated malformations and were all discovered by perinatal autopsy.^6^, ^7^, ^8^ In the remainder of cases, splenogonadal fusion was instead noted as an incidental finding during adolescence or adulthood (age range, 17–74 years).^9^, ^10^, ^11^, ^12^, ^13^ Of these, 2 were discovered intraoperatively during an unrelated surgical intervention,^9^^,^^10^ 2 were found at autopsy,^11^^,^^12^ and 1 at imaging performed for chronic constipation and abdominal pain.^13^ Notably, all the involved ovaries had a normal gross appearance. Normal histology was also disclosed in the 3 ovaries analyzed post-mortem^11^^,^^12^ or after hysteroannessiectomy.^9^ One of the 2 remaining ovaries underwent splenic cord removal at surgery,^10^ thus leaving only one case where splenogonadal fusion was untouched.^13^ All reported female cases were of continuous type, apart from 1 ascertained case of discontinuous type^11^ and one where it was difficult to establish whether a splenic-ovarian cord was present due to extensive pelvic adhesions.^9^ Interestingly, nulliparity was disclosed in 2 cases,^9^^,^^12^ while a motherhood status was indirectly reflected by 2 Cesarean sections acknowledged in the medical history of another case.^11^ Therefore, to our knowledge, the present case represents the youngest living female to be diagnosed with splenogonadal fusion reported to date.

The co-existing genital infantile hemangioma, which accounts for 1 % of all infantile hemangiomas,^14^ was the only associated anomaly encountered in our case. Although the association between facial hemangiomas and splenogonadal fusion has been previously reported,^8^^,^^15^ we speculated that the co-occurrence of two rare conditions, i.e vulvar hemangioma and splenogonadal fusion, was most likely coincidental.

From a diagnostic standpoint, ultrasonography should easily recognize the typical homogenous smooth echotexture of the involved splenic tissue, which may be further characterized by a variety of second-line imaging modalities, including MRI and scintigraphy.^13^

As far as management is concerned, a conservative treatment seems the most appropriate option. Therefore, surgical treatment for splenogonadal fusion should be kept as a last resort, especially if the diagnosis can be made prior to or during surgery. But in all circumstances, surgery should be primarily aimed at preserving the gonad function, thus consisting of merely severing the obstructive band (continuous type) or removing the splenic tissue attached to the gonad (discontinuous type). In the majority of cases, the splenic tissue may be successfully excised without injury to the gonad. However, if splenic and gonadal tissue is intimately fused, there is a serious risk that the gonad is going to be unnecessarily sacrificed.

In males, anecdotal reports have described the association between splenogonadal fusion and testicular germ cell tumors. However, rather than bearing a causal relationship, we concur that such association is merely related to the delayed treatment of a co-existing cryptorchidism.^2^^,^^16^ Notably, no potential risk of malignancy associated with splenogonadal fusion has been reported in females to date, which further underlines the absolute benign etiology of splenogonadal fusion in this subset of patients.

Another potential indication for surgery may be bowel obstruction secondary to extrinsic compression exserted by a continuous splenogonadal fusion form. However, given that the splenic band usually runs along the left paracolic gutter, as also shown in our case, this occurrence seems very unlikely. Nonetheless, Hines and Eggum^17^ reported on a questionable preoperative diagnosis of low-grade mechanical bowel obstruction in a 15-year-old boy. At laparotomy, an elongated projection of splenic tissue was found to compress the splenic flexure of the colon and extend downwards until exiting the abdominal cavity through the left internal inguinal ring. Simple resection of such a cord-like structure relieved the obstruction.

## 4 Conclusion

Splenogonadal fusion is a benign congenital abnormality. Greater awareness among health professionals is paramount to increase splenogonadal fusion identification and, therefore, to prevent unnecessary removal of the involved, but otherwise normal, gonad.

## Declaration of competing interest

The authors declare that they have no known competing financial interests or personal relationships that could have appeared to influence the work reported in this paper.
